# Sigma Factors for Cyanobacterial Transcription

**DOI:** 10.4137/grsb.s2090

**Published:** 2009-04-22

**Authors:** Sousuke Imamura, Munehiko Asayama

**Affiliations:** Laboratory of Molecular Genetics, School of Agriculture, Ibaraki University, 3-21-1 Ami, Inashiki, Ibaraki 300-0393, Japan

**Keywords:** cyanobacteria, gene expression, promoter, σ factor, transcription

## Abstract

Cyanobacteria are photosynthesizing microorganisms that can be used as a model for analyzing gene expression. The expression of genes involves transcription and translation. Transcription is performed by the RNA polymerase (RNAP) holoenzyme, comprising a core enzyme and a sigma (σ) factor which confers promoter selectivity. The unique structure, expression, and function of cyanobacterial σ factors (and RNAP core subunits) are summarized here based on studies, reported previously. The types of promoter recognized by the σ factors are also discussed with regard to transcriptional regulation.

## Introduction

Life depends on organic matter and oxygen produced by photosynthesizing organisms. Cyanobacteria, photosynthesizing organisms capable of producing oxygen, came into being about three billions years ago. It is generally accepted that the ancestor of cyanobacteria gave rise to plant plastids via endosymbiotic events, conferring the photosynthetic ability. Cyanobacteria are Gram-negative prokaryotes that perform oxygenic photosynthesis, found almost all over the earth. There are many kinds of cyanobacteria, including unicellular, filamentous, freshwater, marine, thermostable, and drought-resistant species. Some species are capable of fixing nitrogen, can produce hydrogen, and are possible sources of food. In addition, cells have become fossil fuels. Meanwhile, cyanobacteria have been recognized as a source of numerous natural products^1^ which are structurally interesting bioactive compounds, including toxins, antibiotics, and siderophores. Toxic cyanobacterial waterblooms found in eutrophic lakes, marshes, and dams, are often considered an ecological menace. Thus, the natural variation of cyanobacteria may make them a candidate for a biological catalog, and studies of this organism’s gene expression have special fascination.

Gene expression involves regulation in four steps; transcription, post-transcription, translation, and post-translation. In transcription, the eubacterial RNAP holoenzyme, composed of a σ subunit and a core enzyme containing the major subunits α_2_ (RpoA × 2), β (RpoB), and β′ (RpoC), plays a central role. The core enzyme functions in RNA polymerization and requires the σ subunit for specific transcription initiated at the promoter.^2^,^3^ General switching in transcription is mainly due to the modulated promoter selectivity of multiple RNAP holoenzymes combined with the replacement (“σ switching”) of a common core enzyme with several different σ factors, in response to environmental or internal cellular change.

**Figure 1:**
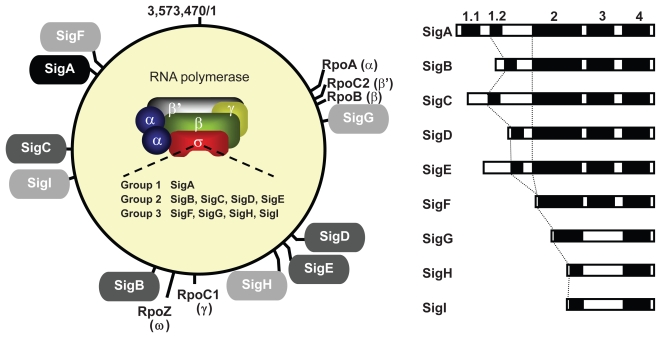
Schematic representation of PCC 6803 RNAP subunits. Positions of the gene encoding the subunits are mapped on the genomic DNA (circle, left panel). The size and conserved regions (black, regions 1 to 4, right panel) among respective σ factors are shown.

The σ factors can be divided into two families, σ^N^ and σ^70^-types, in *Escherichia coli.*^4^,^5^ The RNAP with σ^N^ covers transcription from nitrogen-related genes in *E. coli*. Although cyanobacteria possess a system for nitrogen metabolism, no σ^N^-types exist in cyanobacteria. Also in cyanobacteria, the σ^70^-family can be structurally and functionally subdivided into three groups (Fig. 1). Group 1 comprises a principal σ factor that is essential for cell viability. Group 2 is similar to group 1 in molecular structure, but nonessential for cell viability. Group 3 σ factors are an alternative type, structurally different from the group 1 and group 2 σ factors, and are involved in the transcription of regulons for survival under stress. From a structural point of view, σ factors exhibit conserved domains known as regions 1 and/or 2, 3, and 4 (Fig. 1). Region 1 is subdivided into regions 1.1 and/or 1.2. Region 2 is subdivided into 2.1, 2.2, 2.3, 2,4, and 2.5. Region 2.4 (and 2.5) helps to recognize a −10 (and an extended −10 motif) element. Region 3 is subdivided into 3.1 and 3.2. Region 4 is subdivided into 4.1 and 4.2. Region 4.2 helps to recognize and make contact with a −35 element. X-ray crystallography has resolved the structure of some bacterial RNAPs with σ factors^6^–^10^ revealing the overall shape and size to be similar among enzymes.

## 1. RNA Polymerase Core Enzyme and σ Factors

### 1–1. Cyanobacterial core enzyme

As mentioned in the Introduction, most eubacterial RNAP holoenzymes consist of a core enzyme with subunits of α_2_ββ′(RpoA × 2/RpoB/RpoC). We present a schematic outline for the RpoA (41 kDa) and RpoB (131 kDa) subunits of the cyanobacterium *Synechocystis* sp. PCC 6803 (Fig. 1). The RpoA subunit consists of two independently folded domains, referred to as amino-terminal and carboxyl-terminal domains. The amino-terminal domain is involved in interaction with the other subunits of RNAP. The carboxyl-terminal domain interacts with the template DNA and activators.^11^ RpoB possesses an evolutionally conserved domain, called β-flap, which interacts with the conserved region 4 of the σ factor and triggers a conformational change that moves region 4 into the correct position for interaction with the −35 element.^12^ A characteristic feature of photosynthesizing organisms is that the β′ subunit is usually split into two parts, γ (RpoC1) and β′ (RpoC2). Thus, the composition of the cyanobacterial core enzyme is α_2_ββ′γ (Fig. 1). This architecture is similar to that of almost all plant chloroplasts (α_2_ββ′β″). In order to avoid confusion, it needs to be noted that plant β′ and β″ are encoded by *rpoC1* and *rpoC2*, respectively. The eubacterial β′ (RpoC) subunit, for example of *E. coli*, possesses eight highly conserved domains, A to H,^13^ however, in cyanobacteria and also plant chloroplasts, RpoC1 (67 kDa) and RpoC2 (161 kDa) possess the domains A to D and E to H, respectively.^14^,^15^ Cyanobacterial and chloroplast RpoC2 shows one remarkable structural difference, a large insertion between domains G and H, compared with the same region of *E. coli* RpoC. The insertion is suggested to be a DNA-binding domain as in the jaw module of the largest subunit of RNA polymerase II, RPB1.^16^,^17^ Actually, it has been suggested that the large insertion domain confers the ability to specifically recognize nucleotides on the cyanobacterial promoter *in vivo* and *in vitro.*^17^ The cyanobacterial and eubacterial (*E. coli*)-type core enzymes are essentially the same in molecular structure and function, however, differences in promoter recognition have been observed between them even with the same promoter^17^–^21^ suggesting that the functions of these two core enzymes are not identical. Of note, a small subunit, ω (10 kDa), is not essential for cell viability but contributes to the formation and stability of RNAP by binding to the β′ subunit in *E. coli* or *Thermus aquatics.*^6^,^7^,^22^ Although RNAPs from some cyanobacterial species have been purified from cells, it is not clear whether cyanobacterial RNAP also stoichiometrically contains the ω (RpoZ) subunit, whose gene is usually encoded in the genome (Fig. 1). However, it is assumed that ω is a component of cyanobacterial RNAP. Further functional characterization of core enzyme subunits will provide insight into the fundamental transcriptional mechanism in cyanobacteria.

### 1–2. Cyanobacterial σ factors

#### 1–2–1. Group 1, 2, and 3 σ factors of cyanobacteria

**Figure 2:**
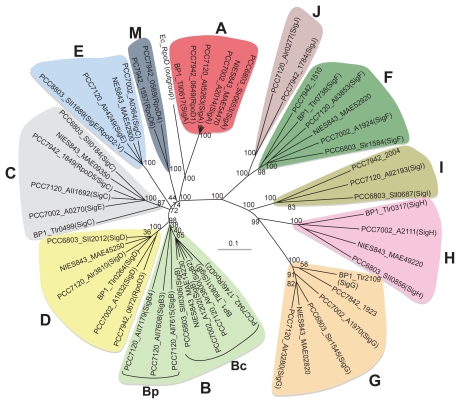
Phylogenetic analysis of group 1, 2, and 3 σ factors of cyanobacteria. The unrooted phylogenetic tree was constructed from the evolutionary distance data^120^ using the neighbor-joining method^25^ with the software ClustalW.^121^ Bootstrap resampling was used to estimate the reliability of the inferred tree. The bootstrap procedure sampled 1,000 times with replacement by ClustalW, and the number at each node represents the percentage of trees supporting the specific branching pattern in the bootstrap analysis. Total amino acid sequences of σ factors from *Synechocystis* sp. PCC 6803, *Synechococcus elongatus* PCC 7942, *Anabaena* sp. PCC 7120, *Synechococcus* sp. PCC 7002, *Microcystis aeruginosa* NIES-843, *Thermosynechococcus elongatus* PB-1, and *Escherichia coli* were used for the phylogenetic analysis as queries. Designations for amino acid sequences of the cyanobacterial σ factors are shown as follows: the “strain name”_“gene number in CyanoBase with or without the named gene product in parentheses”, for example, PCC6803_Slr0653(SigA) indicates *Synechocystis* sp. PCC 6803 Slr0653/SigA. The designation and GenBank accession number for the sequence of *E. coli* (K-12 strain W3100) RpoD is BAE77118. The bars indicate the distances corresponding to 10 changes per 100 amino acid positions. Each name of the proposed subgroups, A to I (with J and M, see sections of 1–2–1 and 2–4), is indicated at the top of relevant clades. Bc and Bp denote *sigB* genes that are encoded in the chromosome, and a plasmid, respectively.

A number of σ factors are encoded by the cyanobacterial genome (and endogenous plasmids) and have been classified into groups 1, 2 and 3, based on phylogenetic analyses.^23^,^24^ The genes encoding the core and σ subunits are scattered in the genome of the freshwater unicellular cyanobacterium *Synechocystis* sp. PCC 6803 (hereafter PCC 6803) (Fig. 1). This time, to assess phylogenetic relationships among σ factors, we performed an analysis using whole amino acid sequences from six major strains whose σ factors have been investigated extensively among cyanobacteria and entire genome sequences (CyanoBase, http://bacteria.kazusa.or.jp/cyanobase/) have been completely determined by the neighbor-joining method.^25^ Results of the analysis are presented in Figure 2 and are summarized in Table 1. The outgroup is *E. coli* RpoD (group 1 σ factor) which has generally been used as a standard σ factor.^2^,^4^,^21^,^24^,^26^–^30^ It is important to note that Figure 2 shows the phylogenetic relationship among some selected cyanobacterial σ factors, however, complete genome sequences of other cyanobacteria including marine cyanobacteria have been elucidated elsewhere.^31^–^33^ The respective cyanobacteria possess a unique group 1 σ factor like other bacterial species, and make a tight monophyletic clade. The group 2 σ factors basically consisted of four distinct clusters, B, C, D and E. In the B cluster, most strains have one protein, but several additional proteins are found in *Anabaena* sp. PCC 7120 (hereafter PCC 7120), which is a filamentous and heterocyst-forming cyanobacterium. These multiple proteins except for Alr3800 (SigB2) are encoded on extra-chromosomal plasmids. Therefore, we subdivided the B cluster into Bc (encoded into a chromosome) and Bp (encoded on plasmids). The C- and D-type σ factors have a single group 2 σ factor in each cluster. In the E clade, this type of σ factor is not found in the freshwater unicellular cyanobacterium *Synechococcus elongatus* PCC 7942 (hereafter PCC 7942), the unicellular thermophilic cyanobacterium *Thermosynechococcus elongatus* BP-1 (hereafter BP-1) and marine cyanobacteria (Fig. 2).^30^ In addition to these clusters, another clade is formed by PCC 7942 RpoD4 and RpoD6. A phylogenetic analysis including σ factors from the six strains and some marine cyanobacteria showed that the PCC 7942 RpoD4 and RpoD6 were assigned to a clade unique to marine cyanobacteria (the M-type as named tentatively in Fig. 2), 30 implying the unique evolutional history of PCC 7942. The group 3 σ factors basically consisted of four distinct clusters, F, G, H and I. In the F and G clusters, almost all strains possess one gene copy, however, PCC 7942 and PCC 7120 possess a second gene copy, which is monophyletic and was recently designated as SigJ (see section 2–4).^29^ All strains analyzed in this study have the G-type σ factors, whereas two or three strains lack H- and I-type σ factors, implying that these two σ factors evolved in a species-specific manner. It is again emphasized that no homologues of the σ^N^ family have been found in cyanobacteria (Table 1). Therefore, it is obvious that the group 1, group 2, and/or group 3 σ factor(s) contribute to the nitrogen-related gene expression in cyanobacteria.

#### 1–2–2. Structure of group 1, 2, and 3 σ factors of PCC 6803

As mentioned in the Introduction, σ factors have evolutionally conserved regions critical for promoter recognition and forming a pre-initiation complex. Next, we summarize the composition and structural features of group 1–3 σ factors of PCC 6803. SigA (type-A) has regions 1 (1.1 and 1.2), 2, 3, and 4. In composition, group 2 σ factors (B, C, D, E-types) are similar to SigA but lack region 1.1, which has been identified upstream of region 1.2^4^,^34^ and is rich in the acidic amino acid residues Asp and Glu. The ratio of acidic amino acids upstream of region 1.2 for the group 2 σ factors together with SigA is as follows: SigA 20/69 (29%); SigB, 1/33 (3%); SigC, 11/47 (23%); SigD, 0/6 (0%); and SigE, 8/55 (15%). Therefore, the ratios of SigC and SigE are relatively high in comparison with those of other group 2 σ factors, implying functional similarity to region 1.1 of principal σ factors. Indeed, both σ factors were found to be closely related in the phylogenetic analysis shown in Figure 2. The group 3 σ factors (F, G, H, and I-types) have regions 2 and 4 (Fig. 1). Among the four-type σ factors, only SigF harbors region 3, however, its similarity with group 1 and 2 σ factors is relatively low. It is conceivable that F-type σ factors play distinct roles among the group 3 σ factors. Actually, G-, H-, and I-type σ factors are expected to act as extracytoplasmic function (ECF) σ factors based on analogy in amino acid sequence with other bacterial ECF σ factors^35^–^37^ that are sometimes called group 4 σ factors in cyanobacteria. The molecular weights of each group also differ greatly corresponding to the differences in structure: group 1, 67 kDa; group 2, 40 to 47 kDa; and group 3, 23 to 30 kDa.^24^ These features are basically conserved in other cyanobacterial strains.

## 2. Expression and Function of σ Factors

In this section, we mainly summarize the research on group 1–3 σ factors in PCC 6803, the most intensively investigated cyanobacterium.

### 2–1. Group 1 σ factors

Eubacterial group 1 σ factors are also known as principal σ factors, and this type σ factor comprehensively contributes to transcription in a cell. The group 1 σ factors are thought to be indispensable for the expression of housekeeping genes, since it is impossible to disrupt their genes. Consistent with this, the levels of the transcript and protein for the PCC 6803 group 1 σ factor, SigA, are constant despite growth phases under normal physiological conditions.^24^ In addition, the group 1 σ factor protein levels are constant under a light/dark cycle and high-light conditions in not only PCC 6803 but also in the freshwater unicellular cyanobacterium *Microcystis aeruginosa* K-81 (hereafter K-81).^24^,^38^,^39^ In contrast, some environmental conditions, heat-shock, darkness, and salt stress, lead to a down-regulation of *sigA* transcription. Concomitantly, the SigA protein level was slightly decreased in heat-shocked cells.^24^ It was also reduced in response to nitrogen deprivation.^40^ These reductions at the protein level are thought to be related to a “σ switching” (see Introduction and section 3).

*In vitro* transcription systems with reconstituted RNAP are a powerful tool for the biological characterization of the promoter selectivity of σ factors. So far, such systems have been established in PCC 6803, PCC7942, PCC7120, and BP-1.^18^–^20^,^24^,^26^ Furthermore, systems using heterologous RNAP, reconstituted with the *E. coli* core enzyme and group 1 σ factors, have been tried for analyses of promoter selectivity in the cases of PCC 6803 and K-81.^38^,^41^–^44^ From these studies with *in vitro* (and also *in vivo*) approaches, group 1 σ factors specifically and efficiently recognize the *E. coli* RpoD consensus type promoters (−35 and −10 elements as TTGACA and TATAAT). The *in vitro* transcripts synthesized by RNAP-SigA (RNAP core enzyme + SigA) of PCC 6803 were undetectable when the mutated −35 element of the *psbA2* promoter (mutated −35 and −10) was used as a template,^20^ indicating that cyanobacterial group 1 σ factors prefer RpoD consensus type promoters similar to other eubacterial group 1 σ factors.

In BP-1, the functional characterization of region 1.1 of SigA has been reported.^19^ In addition to the acidic amino acids in region 1.1, cyanobacterial group 1 σ factors harbor basic amino acids just upstream of region 1.2. Basic amino acids are also observed in the Sig1, Sig2, and Sig6 σ factors of higher plants.^19^,^28^ It has been demonstrated that the eubacterial region 1.1 plays a central role in preventing the nonspecific DNA-binding of group 1 σ factors before binding to the RNAP core enzyme, whereas, interestingly, BP-1 SigA itself has the ability to bind to DNA in a sequence non-specific manner, but this ability is lost upon deletion of region 1.1. These findings provide insight into the novel role of region 1.1 in cyanobacteria, which is distinct from known roles of other group 1 σ factors. However, the functional conservation of region 1.1 in other cyanobacteria and the chloroplast σ factors has not been clarified.

Moreover, it is known that the tertiary architecture of DNA influences gene expression, and sequence-directed (static, intrinsic) or protein-induced DNA bends refer to changes in the DNA double helix. Previous studies presented evidence that a right-handed superhelical curvature upstream of *rpoD1* (a group 1 σ factor gene) Promoter 2 facilitated transcription in K-81.^45^,^46^ Positive regulation also comprises two distinct curved DNAs (CIT and RIB) for K-81 *psbA2* transcription involving RNAP with the group 1 σ factor.^47^–^50^

### 2–2. Group 2 σ factors

#### 2–2–1. SigB-type

##### 2–2–1–1. Heat-shock-responsive SigB

Under heat-shock (42 °C, 30 min) at the mid-exponential phase in PCC 6803, the protein level of SigB significantly increased 12.7-fold compared to that in the control experiment (30 °C). By contrast, the levels of other group 1 and 2 σ factors decreased only slightly or remained constant, and group 3 σ factors were not detectable under heat-shock conditions.^24^ The *sigB* transcripts were also expressed upon the exposure of cells to heat-shock,^24^,^51^,^52^ and heat-shock-responsive transcripts were not observed in the *sigB* knockout strain, indicating that responses to heat-shock involve the autoregulation of *sigB* in PCC 6803.^24^ *hspA* encoding a small heat-shock protein was first identified to be a target of the heat-shock induced σ factor SigB.^24^ Recently, this finding was independently confirmed by two other research groups.^53^,^54^ Moreover, the most effective and productive transcription from the *hspA* promoter consistently observed *in vitro* was by RNAP-SigB among the group 1 and 2 σ factors.^20^ However, the specific promoter-recognition by SigB might be made redundant by other σ factors in this cyanobacterium, since the *hspA* transcript remained even in the *sigB* knockout strain (approximately 40% of the amount in the wild-type strain). No or almost no effect on *hspA* expression by other group 2 type σ factors, except for SigB, was observed under heat-shock conditions. Therefore, the remaining *hspA* transcript in the *sigB* knockout strain may depend on the promoter recognition by SigA. Consistent with this idea, transcription from the *hspA* promoter by reconstituted RNAP-SigA was observed *in vitro.*^20^ In contrast, the heat-shock-induced expression of *groESL1, groEL2, dnaK2,* and *grpA* was not altered in the *sigB* mutant.^24^,^54^ It is plausible that those genes are mainly transcribed by RNAP-SigA and/or another regulatory system as in *Bacillus subtilis.*^24^ Indeed, Singh et al.^53^ recently reported that some key chaperone genes (*groES*, *groEL1*, and *groEL2* but not *hspA*) were negatively regulated by HrcA, which acts as a repressor and prevents the expression of heat-shock proteins by binding directly to CIRCE (Controlling Inverted Repeat of Chaperone Expression).^55^,^56^ On the other hand, a DNA microarray analysis showed that transcription of *htpG*, *hspA, dnaK*, *groEL1*, and *groES* was less induced in the *sigB* knockout strain after temperature upshift (30 °C to 45 °C, 15 min).^53^ The different results obtained with the *sigB* knockout strain may be due to different experimental conditions. However, SigB plays a central role cooperating with SigA/HrcA in the transcription of heat-shock inducible genes. Physiological studies indicated that the *sigB* knockout strain showed retarded growth at 43 °C compared with the parental wild-type strain.^53^ Furthermore, the rate of survival after heat-shock (48 °C, 15 min) was only 2% for cells lacking *sigB*, compared to 20% for wild-type cells.

##### 2–2–1–2. Dark-responsive SigB

The SigB protein level also significantly increased 2-fold after a shift from continuous light to darkness. In contrast, the protein level decreased upon illumination to approximately 25% of that in dark-adapted cells.^39^ Further experiments using two herbicides, DCMU [an inhibitor of electron transport between the PS II complex and the plastoqui-none pool (PQ)] and DBMIB (an inhibitor of electron transport between the PQ and the cytochrome *b_6_* *f* complex), suggested that the oxidative state of the components downstream of the PQ induces SigB synthesis. In darkness, SigB is involved in the regulation of gene expression. The level of dark-induced *lrtA* transcription was not increased in the *sigB* knockout strain.^39^ Consistent with this, a reconstituted RNAP-SigB allowed specific *in vitro* transcription from the *lrtA* promoter.^20^ A recent microarray analysis demonstrated that 160 genes were differently regulated during darkness in the *sigB* knockout strain relative to the wild type,^57^ and 136 of these genes were induced in the mutant, including 28 genes encoding proteins for translation, 10 genes for regulatory functions, 9 genes for photosynthesis and respiration, and 6 genes for energy metabolism. Conceivably, dark-induced SigB expression could be involved in the activation and repression of gene expression for accommodation to darkness and/or in preparation for subsequent exposure to light.

##### 2–2–1–3. Nitrogen starvation-responsive SigB

**Figure 3:**
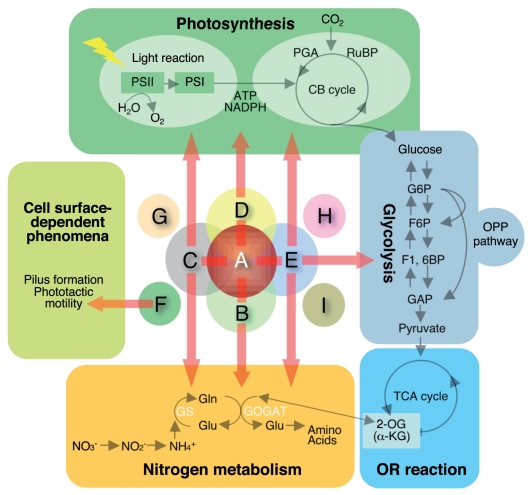
A possible model for functions of PCC 6803 σ factors. Interference of expression among group 1 and 2 σ factors is shown by overlapped circles, A (SigA) to E (SigE). The group 3 σ factor SigF may function independent of the group 1 and 2 σ factors. The functions of SigG, H, and I have not been elucidated in PCC 6803.

Nitrogen deprivation-responsive transcription from the transcription start site of *glnB* (PglnB-54/-53) located at -54/-53 (+1 is the initiation codon), which is regulated by NtcA, a transcriptional activator of the CRP family, is due to specific recognition by SigC in the stationary (post-exponential) growth phase;^58^ see SigC section). The result gave rise to the assumption that another σ factor recognizes the *glnB* promoter in the logarithmic (exponential) growth phase. In the process of identifying the σ factor using group 2 σ factor knockout strains, it was found that SigB specifically contributed to transcription of *glnB* under nitrogen deprivation in the logarithmic phase, but other group 2 σ factors did not. The *glnB* gene expression was also slightly affected in the *sigB* knockout strain in the stationary phase, indicating that SigB is involved in the regulation of *glnB* expression in both phases.^40^ Moreover, we also confirmed the contribution of SigB to the nitrogen-deprivation-responsive transcription of other NtcA-dependent genes, *glnA*, *sigE*, *amt1*, and *glnN*. Consistent with such observations, the SigB protein level was increased approximately 2-fold in response to nitrogen deprivation in both growth phases. Furthermore, transcripts synthesized from the NtcA-dependent *glnB*, *glnA*, *amt1*, and *glnN in vitro* were detected by RNAP-SigB and their levels were significantly increased when NtcA and 2-oxoglutarate (2-OG, a signaling metabolite accumulated under nitrogen deprivation) were added to the reaction mixture.^40^ These results indicated that SigB plays a central role in the NtcA-dependent nitrogen-related gene expression in PCC 6803. Concerning the functional diversity of the PCC 6803 SigB-type σ factor in cyanobacteria, the transcript of the unicellular marine cyanobacterium *Synechococcus* sp. PCC 7002 (hereafter PCC 7002) *sigB*, corresponding to the PCC 6803 *sigB* (Table 1), is specifically expressed in response to nitrogen deprivation.^59^ The PCC 7120 strain possesses four *sigB*-type σ factors in its genome (*sigB2*) and plasmids (*sigB*, *sigB3*, and *sigB4*) (Table 1). Among them, *sigB* and *sigB2* respond to nitrogen deprivation.^27^,^60^ These findings suggest a functional universality and diversity of the SigB-type σ factors in cyanobacteria for nitrogen metabolism (Fig. 3).

##### 2–2–1–4. Multi-functional SigB and its regulation

It has been also reported that transcription of *sigB* is induced in the cell upon exposure to osmotic, salt, or oxidative stress.^61^–^63^ Therefore, SigB is a multi-functional σ factor for manifold environmental stress, and it seems plausible that *sigB* expression is regulated in response to a common signal, as mentioned below. SigB is an autoregulated heat-shock σ factor, which can specifically recognize the *hspA* promoter.^20^,^24^ PCC 7942 HspA plays a central role in ameliorating harmful effects of light during heat-stress through stabilization of the photosystem II (PS II) complex and light-harvesting phycobilisomes.^64^ PCC 6803 *sigB* (about a 5 to 10-fold increase) and *hspA* (about a 3 to 90-fold increase) transcripts are remarkably accumulated upon the exposure of cells to salt- or osmotic-stress, which also leads to inactivation of PS II activity.^61^,^62^,^65^ Furthermore, under conditions of nitrogen deprivation, phycocyanin, the major constituent of phycobilisomes, acts as a source of nitrogen released from degraded phycobilisomes for the synthesis of polypeptides required for acclimation to a new nitrogen status.^66^ Therefore, SigB may be a σ factor sensing the status of the PS II complex and phycobilisomes. Sensing could be realized by measuring the redox state of the electron transport chain in photosynthesis, which varies during nitrogen starvation. The degradation of phycobilisomes is also considered to be useful for minimizing the absorption of excess excitation energy under stressful conditions.^67^ Consistent with this, SigB expression depended on the redox status of the electron transfer chain as mentioned above, and photosynthetic activity decayed faster in the *sigB* knockout strain after heat treatment (48 °C, 15 min) than in the wild type.^54^ Another question is how the cell senses signal transduction. Recent experiments revealed that an osmotic responsive *sigB* transcript was controlled by a two-component regulatory system comprising Hik2 (a sensor histidine kinase, Slr1147) and Rre1 (a response regulator, Slr1783 = Ycf29), and also indicated that an osmotic responsive *hspA* transcript completely disappeared in the *rre1* knockout strain.^62^ Murata’s group also reported that the level of the heat-shock-responsive *sigB* transcript was reduced to less than half at 60 min after heat treatment (34 °C to 44 °C, 20 min) in the wild type, however, it remained high in the *hik34* knockout strain, suggesting that Hik34 (Slr1285) contributes to the repression of *sigB* expression after the transient induction of *sigB* expression. Therefore, it is conceivable that *sigB* expression is controlled by a specific two-component regulatory system, which senses some signal (one candidate is the redox status of the photosynthesis electron transfer chain), and then SigB attempts to adapt to environmental conditions through the up- and down-regulation of gene expression.

The regulatory mechanism for the circadian clock has most extensively been studied in PCC 7942 among the cyanobacterial radiation.^68^ The involvement of PCC 7942 RpoD2 (PCC 6803 SigB-type, Table 1) in the regulation of circadian rhythmic gene expression was first reported in 1996.^69^ In this paragraph, in addition to RpoD2, we briefly refer to the functions of PCC 7942 group 2 σ factors, RpoD5/SigC (SigC-type, Table 1) and RpoD3 (SigD-type, Table 1), for circadian rhythmic gene expression. Tsinoremas et al.^69^ showed that inactivation of *rpoD2* resulted in a low amplitude but still rhythmic expression of *psbAI*. On the other hand, another study indicated that inactivation of *rpoD2* resulted in an elevated level of *psbAI* expression.^70^ Nair et al.^71^ showed that period lengths of *kaiB* (whose gene product functions as the central oscillator for the circadian clock together with KaiA and KaiC) and *purF* (encoding an amidophosphoribosyltransferase) were increased by about 1 hour in the *rpoD2* mutant, whereas amplitude was similar to that of the wild-type strain. Overproduction of RpoD2 increased the period length of *psbAI* by about 2 hours, indicating that RpoD2 is involved in the rhythmic expression in a promoter-dependent manner. They also showed that the expression of *rpoD2* itself peaked at subjective dusk like most genes in PCC 7942, and that RpoD2 negatively regulates its own transcription.^71^ Similar to that of the *rpoD2* mutant, the promoter activity of *psbAI* was elevated by inactivation of *rpoD5*/*sigC.*^70^ However, the period of circadian expression of *psbAI* was prolonged by 2 hours in the *rpoD5*/*sigC* mutant. As for *purF* and *kaiB* expression, both periods were unaffected in the mutant, showing that separate timing circuits with different periods coexist in a cell.^71^ Overproduction of RpoD5/SigC increased the period of *psbAI* expression by about 2 hours as RpoD2 was overproduced. *rpoD5*/*sigC* itself showed a rhythmic peak in expression at subjective dusk as *rpoD2* did, but the amplitude of its promoter activity was higher than that of *rpoD2.*^71^ As for *rpoD3*, its deletion resulted in about a 50% decrease in the amplitude of *psbAI* expression and a shift in phase by ~4 hours.^71^ The period of *purF* expression was prolonged by about 3 hours and its amplitude was decreased in the mutant. On the other hand, the expression of *kaiB* was not significantly affected in the *rpoD3* mutant. Therefore, the *rpoD3* mutation affected promoter activities in three different ways. Interestingly, although overproduction of RpoD3 resulted in arrhythmic expression of *psbAI,*^71^ the expression of *kaiB* was not affected. The expression of *rpoD3* rhythmically peaked at subjective dusk as for *rpoD2* and *rpoD5/sigC.*^71^ In PCC 6803, although it has been unclear which σ factors function in circadian rhythmic gene expression, it seems plausible that SigE plays an important role (see section 2–2–4).

#### 2–2–2. SigC-type

##### 2–2–2–1. Physiological roles of SigC

In the process of investigating the function of SigC, Asayama et al.^58^ found that the growth of *sigC* knockout cells was slightly reduced and viable cell counts on BG11 plates were significantly lower (3.2 × 10^6^ ml) in the stationary phase, compared with the value for wild-type cells (7.8 × 10^7^ ml). The reduced cell viability in the *sigC* mutant at the stationary phase was also confirmed by trypan blue staining. Furthermore, they also confirmed that the number of lysed cells was larger in the mutant than wild-type culture by transmission electron microscopy. It was recently reported that differences in cell density between the wild-type and *sigC* knockout strain after prolonged heat treatment can be partially ascribed to differences in the stationary phase between these strains.^72^ The results clearly indicated that *sigC* is a group 2 type σ factor, which is effective for cell viability in the stationary phase.

##### 2–2–2–2. Stationary-specific nitrogen gene expression by SigC

A proteome analysis using two-dimensional SDS-PAGE with total protein prepared from the *sigC* knockout strain in the stationary phase under white-light illumination revealed that the signal corresponding to a nitrogen regulatory gene (*glnB*) product, PII, was significantly reduced in intensity in the *sigC* mutant^58^ (Fig. 3). PII, a signal transduction protein conserved among bacteria, archaea and plants, senses the nitrogen status, and is also considered to play pivotal roles in monitoring the carbon/nitrogen balance.^73^ A subsequent Western blot analysis using a specific antibody against GlnB demonstrated that the protein level of GlnB was markedly reduced in *sigC* knockout cells under nitrogen depletion at the stationary phase, but not logarithmic phase of growth. Moreover, results obtained by transcriptome using high density gene filters (HDF, macroarray filter) and quantitative real time (QRT)-PCR analyses revealed a decrease in the *glnB* transcript in the *sigC* knockout strain at the stationary phase, but not logarithmic phase. These results showed that *glnB* transcription specifically depends on SigC at the stationary phase. Further high-resolution analysis of the contribution of SigC to *glnB* expression from the two transcription start sites (PglnB-54/-53 and -33)^74^ demonstrated that nitrogen deprivation-responsive transcription from PglnB-54/-53 in the *sigC* mutant at a mid-log phase of growth was equal to that in the wild type, whereas a significant induction in response to nitrogen deprivation was not observed in the *sigC* knockout strain. The inactivation of *sigC* did not affect the transcription from the PglnB-33 promoter.^58^ With respect to other nitrogen-regulated genes, QRT-PCR analyses also revealed that transcript levels of *glnA*, *amt1*, and *sigE* were decreased in the *sigC* knockout strain under nitrogen deprivation during the stationary phase, but not affected during exponential growth. Consistent with these findings, *in vitro* transcription analyses using reconstituted RNAP-SigC demonstrated that SigC specifically and effectively recognized NtcA-dependent promoters in the presence of NtcA and 2-OG.^40^,^58^ Thus, SigC widely regulates nitrogen-regulated genes during the stationary phase under conditions of nitrogen deprivation.^58^ These findings indicated that the NtcA-dependent nitrogen-related genes are mainly recognized by SigB during exponential growth (see above) and by SigC at the stationary phase in response to nitrogen deprivation, namely, a “σ-switch” for those recognizing nitrogen promoters corresponding to the growth phase. It is worth noting that a difference in specificity between SigB and SigC for the *glnN* promoter was observed *in vivo* and *in vitro.*^40^ A previous study reported that the control mechanisms for PCC 6803 *glnA* and *glnN* are essentially different.^75^ The authors suggested that an additional modification of NtcA or additional factor was required for the activation of *glnN*, and pointed out possible regulation by a palindromic inverted repeat sequence in the region upstream of the NtcA-binding motif of *glnN* (−130 to −88, +1 is the initiation codon). The lack of recognition of the *glnN* promoter by SigC may be caused by the unique structure in addition to the low similarity of NtcA-binding motifs of *glnN* among the NtcA-dependent promoters.^40^,^75^ In PCC 7002, *sigE*, corresponding to PCC 6803 *sigC* (Table 1), is required for gene expression during the post-exponential growth phase but its mRNA decreased after the mid-exponential phase.^76^ These findings suggested that the PCC 6803 SigC-type σ factor is evolutionally conserved in cyanobacteria and may function as a key protein for stationary-specific gene expression to acclimate and coordinate cellular processes involving the assimilation of nitrogen.

Primer extension analyses revealed that the *sigC* gene possesses three putative transcription start points, among which transcripts from PsigC-38 and -31 were increased and those from PsigC-141/-140 remained constant with cell growth.^24^ On the other hand, the amount of SigC protein in the stationary phase was almost half that in the log phase, and the amount was almost constant even when nitrogen was depleted.^24^,^40^ This raises the question of whether SigC is specifically activated in the stationary phase. Possible explanations include (i) a post-translational regulatory effect (e.g. anti-σ factor, phosphorylation and/or other modifications), and (ii) a guanosine-3, 5-(bis)pyrophosphate (ppGpp) effect, reported in some bacteria,^77^–^80^ though none of these hypotheses has been clarified, nor has the regulation of *sigC* expression in response to environmental conditions except for the regulation by other σ factors (see above and below).

In PCC 7120, NtcA and FurA, which is the ferric uptake regulator protein for iron homeostasis, bind to the promoter region of *sigC*, implying that PCC 7120 SigC contributes to nitrogen metabolism in connection with iron homeostasis.^81^ In PCC 7942, use of the *gfp* transcript to investigate the temporal and spatial pattern of expression of *sigC* (PsigC-gfp) revealed a rapid increase of GFP fluorescence in the *sigC* reporter strain 4 hours after a nitrogen step down (from a nitrate-plus medium to nitrate-free medium),^82^ implying that a functional conservation of SigC-type σ factors as well as SigB-type σ factors (see above) is involved in nitrogen metabolism in cyanobacteria that do not possess the *E. coli* σ^N^-type σ factor (Table 1).

##### 2–2–2–3. SigC for heat acclimation

Recently, Tuominen et al.^72^ characterized the PCC 6803 *sigC* knockout strain under conditions of heat acclimation. The strain showed a low survival rate and poor growth compared to the wild-type strain under heat-stress conditions in liquid cultures. Under the stress, the amount of *sigA* transcript decreased in both the knockout and wild-type strains. But this decrease was more rapid and prominent in the mutant strain. Thus, they discussed that the reduced growth is related to the inability of the *sigC* knockout strain to produce normal amounts of the *sigA* transcript under heat stress. However, it has not been demonstrated whether SigA protein actually decreased in proportion to the level of *sigA* transcript in the mutant. Subsequently, they found that the equilibrium concentration of CO_2_ at 43 °C was 76% of the concentration at 32 °C when cells were grown in air. Therefore, they examined the role of CO_2_ in the acclimation to high temperatures by growing the wild-type and the *sigC* knockout strain at 43 °C in air supplemented with 3% CO_2_. The result showed that the change from an ambient air supply to air supplemented with 3% CO_2_ enhanced the growth of the *sigC* knockout strain even under heat stress. A genome-wide DNA microarray analysis revealed differences in the expression of many genes related to carbon-concentrating mechanisms between the wild-type and the *sigC* knockout strain. On the other hand, consistent with a previous study, (See section 2–2–3–1)^24^ the heat-shock-responsive *groESL* and *hspA* transcripts were not altered in the *sigC* knockout strain compared with the wild-type strain. Thus, the inability of the *sigC* knockout strain to grow at high temperature in normal air with CO_2_ is partly dependent on the poor availability of inorganic carbon at high temperature and partly on another, yet unknown, factor.

#### 2–2–3. SigD-type

##### 2–2–3–1. SigD expression and its regulation

In 2003, Imamura et al. examined fluctuations in the protein levels of all σ factors under high-light conditions in PCC 6803 (transition from 35 to 120 μmol photons m^−2^ s^−1^), and first identified a σ factor, SigD, whose protein was significantly accumulated in response to the change in light intensity^24^ (Fig. 3). At the mid-exponential phase, the level of SigD specifically increased to 3.4-times that under normal light, but levels of SigA and other group 2 σ factor were not elevated under the same conditions.^24^ Subsequently, the protein level of each σ factor in light (35 μmol photons m^−2^ s^−1^) or darkness was also examined by Western blot analyses.^39^ When PCC 6803 cells were exposed to light after adapting to darkness, the SigD level significantly increased at 1 hour (about a 2.5-fold increase compared with 0 hour) and this persisted for 9 hours. Interestingly, the increase of SigD caused by the dark-to-light shift was enhanced in the presence of DCMU or DBMIB, suggesting that the “reductive state of the components upstream of the PQ” induces SigD synthesis. Thus, these results together with those for SigB (see below) indicated that SigB and SigD are antagonistic functional σ factors acting in response to changes in light, the expression of which depends on the redox state of the electron transport chain of photosynthesis (oxidation or reduction). As for the transcript of *sigD*, Hihara et al.^83^ found that the mRNA level was increased by high-light exposure (transition from 20 μmol photons m^−2^ s^−1^ to 300 μmol photons m^−2^ s^−1^) in a genome-wide analysis with a DNA microarray. On the other hand, light-induced SigD synthesis was observed in the presence of rifampicin (inhibitor of transcription) but not chloramphenicol (inhibitor of translation),^39^ indicating that the light-induced expression of SigD was mainly achieved at the post-transcriptional level. Recently, it was reported that PCC 7942 *rpoD3*, corresponding to PCC 6803 *sigD* (Table 1), accumulated under high-light conditions (transition from 35 to 1,500 μmol photons m^−2^ s^−1^) at the level of the transcript and protein. This accumulation is specific to high-light, since *rpoD3* expression was not significantly altered in cells exposed to high salt (0.5 M NaCl), high osmolarity (0.5 M sorbitol), darkness, high temperature (42 °C), or low temperature (22 °C).^84^ They also indicated the post-transcriptional regulation of RpoD3 expression. This mechanism seems to be similar to PCC 6803 SigD as mentioned above. The *rpoD3* deletion mutant rapidly lost viability under high-light conditions. Therefore, the PCC 6803 SigD-type σ factor is evolutionally conserved in cyanobacteria and may function as a key protein for light- and high-light-responsive gene expression in response to changes in light.

DNA microarray analyses showed that transcription of PCC 6803 *sigD* was induced by several environmental factors other than light, including low temperature (transition from 30 °C to 22 °C), heat-shock (transition from 30 °C to 45 °C), salt stress (0.5 M NaCl), high osmolarity (0.5 M sorbitol), and hydrogen peroxide (0.25 mM H_2_O_2_).^53^,^63^,^85^,^86^ However, Tuominen et al.^52^ performed a Northern blot analysis and found that the *sigD* transcript did not accumulate under heat-shock (transition from 32 °C to 42 °C), low temperature (transition from 32 °C to 16 °C), and high salt (0.5 M NaCl). The different results might be due to different experimental conditions and detection methods. In the normal physiological growth phase, *sigD* transcripts starting from three putative transcription start TSPs (PsigD-25, -22, and -17) were detected at almost constant levels, but the protein level of SigD was higher in the exponential phase than stationary phase.^24^

Some regulatory factors for *sigD* transcription were identified by Murata’s group. They showed that the mutation of *hik33*, encoding a well-known sensory histidine kinase involved in multiple stress responses, led to reduced *sigD* induction by osmolarity stress or cold-shock.^62^,^86^ The osmolarity stress-responsive *sigD* transcript was also reduced by knockout of *rre31*, a response regulator gene, indicating that the Hik33-Rre31 two-component system transduces osmotic signals for *sigD* transcription.^62^ In PCC 7942, Seki et al.^84^ recently revealed that phosphorylated RpaB (as an ortholog of Rre26 = Slr0947 = Ycf27 in PCC 6803), the response regulator of the two-component system,^87^ repressed *rpoD3* (as the *sigD* type of PCC 6803) transcription by binding to the HLR1 (high light-responsive element 1) motif, located just downstream of the TSP of *rpoD3*, under normal growth conditions, and the dephosphorylation of RpaB induced by high-light stress results in transcriptional derepression of *rpoD3*. However, the mechanism by which light-sensing signal transduction, involving changes in the redox state of the electron transport chain in photosynthesis, is linked to the light-responsive transcription with σ factors has been unclear. In PCC 7942, results from a recent study indicated that the SasA (as an ortholog of Hik8 in PCC 6803) –RpaA (Rre31 in PCC 6803) two-component regulatory system functions as a major circadian timing mediator.^88^ In PCC 6803, it has been unclear whether Hik33-Rre31 (and/or Hik8-Rre31) also functions as an integral circadian timing mediator for the expression of genes including *sigD*, and Rre26 is implicated in the regulation of *sigD* transcription under high-light as in PCC 7942. Further studies are needed to clarify the regulatory mechanism of *sigD* expression in response to environmental stress.

##### 2–2–3–2. Genes controlled by SigD

In 2003, it was reported that the light-induced σ factor PCC 6803 SigD specifically recognized the promoters of photosynthetic genes, *psbA2* and *psbA3*, and contributed to their light-induced transcription.^39^ Since then, it has been shown that SigD also contributes to the transcription of the light-induced photosynthetic genes *cpcBADC*, *petBD*, and *psaAB*. This demonstrates a universal function of SigD for light-induced transcription in cyanobacteria^39^,^89^ (Fig. 3). Recently, a genome-wide microarray analysis using a *sigD* knockdown strain was conducted.^57^ In light, some 345 genes displayed transcript levels different from those in the wild type, with 214 of these genes up-regulated in the mutant. The genes with elevated transcript levels included those encoding proteins involved in photosynthesis and respiration (22 genes), amino acid biosynthesis (15 genes), energy metabolism (12 genes), regulation (12 genes), and translation (12 genes). The genes with reduced transcript levels in the *sigD* mutant observed by Imamura et al. and Yoshimura et al. (see above) were not detected as genes with reduced levels in the microarray analysis. As they discussed, the difference may be due to the dynamic range of their system, in which the detection of an ~1.5-fold reduction (observed by Imamura et al. and Yoshimura et al.) is difficult.

#### 2–2–4. SigE-type

##### 2–2–4–1. SigE expression and its regulation

The characterization of PCC 6803 SigE was first reported in 2001.^90^ Muro-Pastor et al. carried out a search in silico for a target sequence, GTAN_8_TACN_21_TAN_3_T, bound by NtcA (a nitrogen controlled activator), and identified it approximately 270 nucleotides upstream from an initiation codon (ATG, A is +1) of *sigE* (*rpoD2-V*) in the PCC 6803 genome. Northern blot analysis indicated that transcription of *sigE* was indeed induced in the nitrogen-deprived cells. Further 5′-end mapping of the *sigE* transcript by primer extension showed that the transcription from PsigE-202 was constitutive, whereas the transcription from PsigE-264 increased with nitrogen deprivation. This suggested that the transcription from PsigE-264 was controlled by NtcA. Actually, a gel-shift analysis confirmed that purified NtcA bound to the *sigE* promoter.^90^ Moreover, it was shown that *sigE* expression at the level of the transcript and protein was increased by nitrogen depletion in a NtcA-dependent manner.^91^ Under normal growth conditions, the transcript from PsigE-202 was expressed at almost constant levels, but that from PsigE-264 was not detected despite the growth phase.^24^ Thus, NtcA is a positive regulator for *sigE* expression through the induction of transcription from PsigE-264 in response to nitrogen deprivation. On the other hand, the transcription from PsigE-202 is thought to be controlled by the group 1 σ factor, SigA, since the transcript was not affected by disruption of each group 2 σ factor gene.^40^,^58^ As for SigE-type σ factors in other cyanobacteria, the transcript of PCC 7002 *sigC* (corresponding to PCC 6803 *sigE*, Table 1) increased under nitrogen deprivation.^59^ In PCC 7120, a bioinformatic analysis predicted a NtcA-binding site within the *sigE* promoter region, raising the possibility that NtcA regulates the transcription of *sigE* in PCC 7120 the same as in PCC 6803.^92^ Recently, it was suggested that PCC 7120 SigE is involved in the expression of late-stage heterocyst-specific genes based on a PsigE-gfp reporter analysis.^82^ Therefore, it seems plausible that SigE-type σ factors are involved in the regulation of nitrogen metabolism (Fig. 3).

In 1997, light-responsive transcripts of K-81 *rpoD2* (the *sigE*-type of PCC 6803) were found as the first case of σ factor genes in a cyanobacterium.^93^ It was also reported that SigE expression fluctuated under light/dark conditions. When the PCC 6803 cells were exposed to light after adapting to darkness, the amount of SigE gradually increased after 3 hours, and reached a peak at 9 hours (approximately 10 times that at 0 hour), suggesting that SigE is a slow responsive light-induced σ factor. In contrast, the amount of SigE decreased in darkness, the level at 9 hours being approximately 65% of that at 0 hour.^39^ In addition, a genome-wide analysis revealed that the PCC 6803 *sigE* expression exhibits a circadian rhythm at the transcript level.^94^ Recently, Yoshimura et al.^89^ investigated the rhythmicity of the expression of PCC 6803 SigE at the protein level under continuous light following a dark (12 hours) resetting. Interestingly, the protein level had an amplitude of rhythm (approximately 24 hours), and peaked every 12 hours, a timing which is somewhat delayed compared to that of its transcript.^94^ These results indicated that *sigE* expression at the level of the transcript and protein is regulated by the circadian system.

##### 2–2–4–2. Genes controlled by SigE

Since *sigE* was identified as encoding a nitrogen responsive σ factor, the transcript levels of nitrogen-regulated genes, *amt1*, *glnB*, and *glnN,* in the *sigE* knockout strain were examined.^90^ A Northern blot analysis showed that the transcript of *amt1* or *glnB* was not significantly altered in the mutant, however, activation of the transcription of *glnN* was impaired in the mutants; a decrease of about 30% was observed after 4 hours of nitrogen starvation, and recently, the same results were also obtained by Asayama’s group.^40^ Subsequently, a genome-wide DNA microarray analysis was performed to identify genes controlled by SigE under normal physiological conditions.^95^ The down-regulated genes included several whose products contribute to sugar catabolism, including enzymes that participate in glycolysis [*pfkA* (encoding one of two phosphofructokinases), *gap1* (one of two glyceraldehyde-3-dehydrogenases catalyzing catabolic reactions)*, pyk1* (one of two pyruvate kinases)], the oxidative pentose phosphate (OPP) pathway [*zwf* (a glucose-6-phosphate dehydrogenase, G6PD), *opcA* (a positive regulator for G6PD), *gnd* (6-phosphogluconate dehydrogenase, 6PGD), and *tal* (transaldolase)], or glycogen breakdown [*glgX* (one of two glycogen isoamylases) and *glgP* (one of two glycogen phosphorylases)]. Consistent with the analysis of the transcripts, the *sigE* knockout strain showed a reduced rate of glucose uptake and an increased intracellular level of glycogen. Moreover, the mutant was unable to proliferate under light-activated heterotrophic growth conditions. These results clearly indicate that SigE functions in the transcriptional activation of sugar catabolic pathway-related genes in PCC 6803 (Fig. 3).

It was also examined whether SigE is implicated in light-responsive gene expression, since SigE protein was also accumulated under light as mentioned above.^39^,^89^ The levels of light-responsive transcripts of *psbA*, *cpcBADC*, *petBD*, and *psaAB* were relatively low in the *sigE* knockout strain, compared to the wild-type strain.^89^ It was also demonstrated that SigE could directly recognize those promoters in *in vitro* transcription analyses with reconstituted RNAP-SigE, indicating that SigE also contributes to the light-induced gene expression (Fig. 3).

For light-responsive gene expression, nonribosomal peptide synthetase genes responsible for the biosynthesis of microcystin (*mcy*) and micropeptin (*mip*) have been identified and characterized in the freshwater unicellular cyanobacterium *Microcystis aeruginosa* K-139.^96^–^99^ Light-induced transcripts of *mcyA* and *psm* (peptide synthetase of *Microcystis*) *3A*/*3I* were revealed and their possible promoters exhibited the consensus sequences recognized by the *E. coli* RpoD-type σ factor. Do σ factors such as the SigA-, SigD-, and SigE-types contribute to light-induced gene expression? This is an interesting question because nonribosomally synthesized peptides produced by some bacterial and fungal species belong to a diverse family of natural products that include antibiotics, immunosuppressants, plant and animal toxins, and enzyme inhibitors for which mechanisms of biosynthesis and light-responsive gene regulation have yet to be elucidated.

##### 2–2–4–3. Physiological roles of SigE

For photosynthesizing organisms, the carbon (stored as starch) produced by photosynthetic reactions is utilized as a source of energy through catabolic pathways in the dark (night). Actually, the expression of genes involved in the catabolism of sugar showed a circadian rhythm, which peaked during darkness (at 11 to 14-hour intervals).^94^ Some such genes were induced to express by nitrogen depletion.^91^ The expression of sugar catabolism-related genes in response to nitrogen deprivation was also observed in non-diazotrophic and diazotrophic cyanobacterial strains.^100^–^102^ Therefore, these phenomena seem to be conserved in cyanobacteria and the possibility exists that the OPP pathway provides reducing power for either respiratory electron transport or nitrogenase during nitrogen fixation in diazotrophic cyanobacteria (Fig. 3). As mentioned above, SigE specifically contributes to the expression of genes related to sugar catabolism and photosynthesis, and is regulated by the circadian system at the transcript and protein levels. Therefore, SigE may be a σ factor controlling the balance of carbon and nitrogen metabolism with a rhythmic expression that peaks at 24-hour intervals according to the upcoming night.^89^ For more information on SigE and the regulation of sugar catabolism by light- and nitrogen-status, please refer to a recent review.^103^ As mentioned above, SigE-type σ factors are not found in PCC 7942, BP-1 and some marine cyanobacteria (Table 1), suggesting that another type of σ factor compensates for the functions of SigE in these cells.

### 2–3. Group 3 σ factors

#### 2–3–1. SigF-type

**Figure 4:**
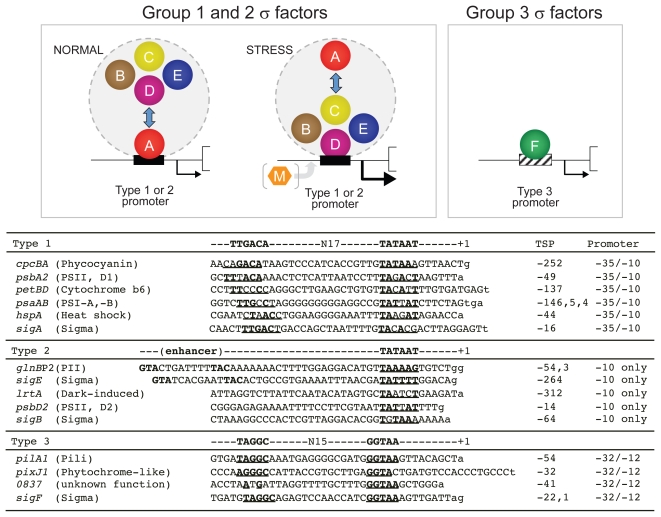
Promoter types of PCC 6803. Promoters recognized by RNAPs with group 1 and 2, or group 3 σ factors are shown (top). Nucleotide sequences for representative type 1, 2, and 3 promoters are presented with transcription start points (TSP, as +1 for the start codon of each structural gene). Details are described in the text.

Among the nine σ factors of PCC 6803, SigF was the first to have its function elucidated in 1999, and is the most intensively investigated group 3 σ factor in cyanobacteria. Bhaya et al.^104^ created a *sigF* knockout strain and found a pleiotropic phenotype. Most notably, the knockout strain lost phototactic movement with a concomitant loss of pili, which are abundant on the surface of wild-type cells (Fig. 3). Furthermore, the *sigF* knockout strain had dramatically reduced levels of transcripts from two tandemly arranged *pilA1* and *pilA2* genes, which encode major structural components of type IV pili. It was indicated that SigF plays a critical role in motility by controlling the formation of pili and is also likely to regulate other features of the cell surface. However, in that study, it was still unclear whether SigF directly regulates *pilA1A2* gene expression or whether the marked reduction in the level of the *pilA1A2* transcript is the outcome of a global effect of SigF on the cell surface architecture. Recently, direct recognition of the PCC 6803 *pilA* promoter by SigF was confirmed by genetic and biochemical analyses.^21^ The 5′-end mapping of the *pilA* transcript by primer extension revealed a single transcription start point (+1), from the promoter PpilA1-54, in wild-type cells. The *pilA* transcript from PpilA1-54 completely disappeared in the *sigF* knockout strain, but not on the knockout of group 2 and group 3 σ factors. Further *in vitro* transcription analysis using each reconstituted RNAP with one of the nine σ factors clearly demonstrated that only SigF is capable of recognizing the *pilA* promoter. The transcription of *sigF* from PsigF-22/-21^24^ observed in the wild type was not detected in the *sigF* knockout strain.^21^,^105^ Consistent with this finding, *sigF* transcription was specifically driven *in vitro* by reconstituted RNAP-SigF, indicating that *sigF* expression is autoregulated.^21^ These results clearly indicated that autoregulated SigF stringently recognizes the *pilA1* promoter in PCC 6803. Furthermore, *in vitro* transcription analyses using scanning mutagenized template DNAs of PpilA1-54 identified the region from −39 to −7 including an AG-rich stretch and a core promoter with TAGGC (−32 region) and GGTAA (−12 region) as important for transcription. The core promoter of PCC 6803 *pilA1* is identical to that of PCC 6803 *sigF* (Fig. 4) and similar sequences were also observed upstream of other *pilA* genes in cyanobacteria, *Gloeobacter violaceus* PCC 7421, BP-1, PCC 7120, and *Synechococcus* PCC 6301. A bioinformatic analysis based on the identified core promoter found more than 50 genes as candidates for promoters recognized by SigF. Subsequently, two of them, *sll0837* (encoding a periplasmic protein of unknown function) and *sll0041* (phytochrome-like phototaxis protein, histidine kinase of a two-component system, PixJ1 = PisJ1 = TaxD1, Tsr or CheD homologue), were experimentally verified to be targets of SigF, their promoter sequences being well similar to the identified SigF recognition core promoter (Fig. 4). Thus, the specificity of SigF-type σ factors could be conserved and distinct compared with those of group 1 and 2 σ factors in cyanobacteria (Fig. 4). It was also revealed that *E. coli* σ factors could not recognize the *pilA1* promoter in *E. coli* cells. Actually, the sequences of core promoters recognized by PCC 6803 SigF were not similar to those recognized by σ factors that are functional counterparts of PCC 6803 SigF, including *E. coli* RpoF, *Pseudomonas aeruginosa* RpoF, and *B. subtilis* SigD.^106^–^108^ A phylogenetic analysis showed that PCC 6803 SigF was assigned, not to the same node as *E. coli* RpoF and *B. subtilis* SigD, but to *B. subtilis* SigB^108^ which contributes to a general stress response. This finding is consistent with a previous study in which the PCC 6803 *sigF* promoter was of the *B. subtilis* SigB type.^24^ *B. subtilis* SigB can recognize a promoter, RGGXTTRA-N14-GGGTAT, the sequence of which is partially similar to that at the −12 promoter of PCC 6803 SigF. These findings strongly suggest that in structure and/or selectivity, PCC 6803 SigF is a novel type of eubacterial group 3 σ factor.

The physiological characterization of the PCC 6803 *sigF* knockout strain was also investigated by Hagemann’s group.^36^ Alterations of gene expression were analyzed in salt (684 mM NaCl, 30 min)-, heat (30 °C to 48 °C, 30 min)- and highlight (170 μmol photons m^−2^ s^−1^ to 2,000 μmol photons m^−2^ s^−1^)-treated cells by *in vivo* labeling of proteins with [^35^S]methionine and subsequent electrophoretic separation of soluble proteins from cell lysates. Results from one- and two-dimensional electrophoresis indicated that the *sigF* knockout strain exhibited markedly decreased levels of salt-induced *de novo* protein synthesis. The reduction was not observed under the other conditions, but one (approximately 16-kDa protein) of the proteins was also decreased in the high-light-treated cells. The tolerance of the *sigF* knockout strain for 684 or 767 mM NaCl was similar to that of the wild type, whereas 859 mM NaCl was lethal to the mutant. It was also found that exposure of cells to high-light (1,500 μmol photons m^−2^ s^−1^ for 2–3 h) was lethal to the *sigF* knockout strain. However, the mechanism by which SigF is involved in salt- and high-light-related gene expression is totally unclear.

In PCC 7002, the *sigF* knockout strain showed much slower growth than the wild type when the cells grown at 38 °C were exposed to 15 °C.^37^ At 22 °C, the *sigF* mutant had the same growth rate as the wild type, suggesting that *sigF* is required for the cells to grow at 15 °C but not 22 °C. However, the mechanism has remained obscure.

So far, endogenous PCC 6803 SigF protein has not been detected under normal physiological conditions or various stressful conditions by Western blotting using an antibody which specifically recognizes recombinant SigF protein;^24^ (Imamura and Asayama, unpublished data). The situation is the same for other PCC 6803 group 3 σ factors, SigG, SigH, and SigI, suggesting group 3 protein levels to be extremely low but sufficient for them to function.

#### 2–3–2. SigG-type

In PCC 6803, the *sigG* gene is essential for cell growth, since a completely segregated *sigG* mutant has not been obtained.^36^,^105^ Consistent with this, the *sigG* knockout strain showed a decreased growth rate in spite of its incomplete segregation.^36^ It was also found that exposure of the *sigG* knockout strain to about 1,500 μmol photons m^−2^ s^−1^ for 2–3 hours at 29 °C was lethal. A primer extension analysis revealed that transcripts of *sigG* were synthesized with three putative transcription start sites (PsigG-34, -26, and -16) and increased dependent of the growth phase.^24^ The sequence of PsigG-34 exhibits similarity to that of the *E. coli* promoter recognized by RpoE (σ^24^). On the other hand, PsigG-26 and -16 exhibit the sequence-type recognized by *B. subtilis* σ^B^.

The necessity of *sigG* for cell growth differs between PCC 6803 and PCC 7002 because disruption of the gene was feasible in PCC 7002.^37^ Products of genes downstream that may be associated with the ECF σ factor genes are anti-σ factors that bind directly to the σ factors to inhibit their activities in *E. coli, P. aeruginosa*, and *Myxococcus xanthus.*^109^ Northern blot and RT-PCR analyses revealed that *sigG* and a gene downstream of it, *sapG* (*sigG*-associated protein), are cotranscribed.^37^ A yeast two-hybrid analysis demonstrated that the *sigG* and *sapG* gene products interact when produced in yeast cells. The *sigG* or *sapG* mutant strain grew as well as the wild-type strain at 38 °C, indicating that these genes are not required for cell growth under optimal conditions. In contrast, the *sigG* mutant could not grow continuously at 22 °C, and could not grow at all at 15 °C. The *sapG* mutant showed a similar growth phenotype, suggesting that SapG is a regulatory protein for SigG involved in the same pathway for acclimation to low temperature. Interestingly, the structure of *sigG*-*sapG* in the cyanobacterial genome is conserved in PCC 6803, PCC 7942, PCC 7120, BP-1, and NIES-843 (CyanoBase). It is thus conceivable that the action of SapG toward SigG is conserved in cyanobacteria.

In PCC 7120, the expression of *sigG* occurred between 9 and 13 hours after a nitrogen step-down according to PsigG-gfp reporter analyses,^82^ when cells become committed to completing the differentiation process, suggesting SigG to be involved in the mechanism of commitment.^110^ The genes controlled by SigG-type σ factors have remained to be elucidated in cyanobacteria.

#### 2–3–3. SigH-type

The 5′-end mapping of transcripts for the *sigH* gene was performed by primer extension analysis, and two putative transcription start points were identified (PsigH-73 and -36/-35).^24^ The levels of transcription from PsigH-73 and -36/-35 were increased and almost constant dependent on the growth phase, respectively. It was also found that PsigH-73 and -36/-35 contain the *E. coli* RpoE (σ^24^) and *B. subtilis* σ^B^ recognition promoter, respectively. Huckauf et al.^36^ found that the expression of the *sigH* gene was induced 7 hours after heat shock (from 30 °C to 43 °C). At this time, however, the transiently activated expression of the heat-shock gene *groEL* was already reduced nearly to the control level. Thus, it is unlikely that SigH is responsible for the quick heat-shock-responsive transcription. But there is no information available regarding genes controlled by SigH-type σ factors in cyanobacteria.

#### 2–3–4. SigI-type

Information is still limited about SigI-type σ factors. In PCC 6803, a primer extension analysis revealed that *sigI* possesses two putative transcription start points (PsigI-100 and -50) that contain the *B. subtilis* σ^B^ recognition promoter, and the transcript levels increased with cell growth.^24^ The PCC 6803 *sigI* gene is not essential for cell growth, since a completely segregated *sigI* mutant has been obtained.^105^

In PCC 7120, the PsigI-gfp reporter strain showed that the *sigI* promoter region (206 bp upstream of the *sigI* coding region) was active in vegetative cells and heterocysts.^82^

### 2–4. Other-type σ factors

As mentioned above, the result of a phylogenetic analysis indicated that some cyanobacterial group 2 σ factors compose independent clusters besides SigB-, SigC-, SigD, and SigE-types (see section 1–2–1). Some of their σ factors are classified into the M-type which is divided into four subtypes, M1 to M4,^30^ generally found in marine cyanobacteria. In PCC 7942, there are two M-type σ factor genes, *rpoD4* (M1-type) and *rpoD6* (M2-type) (Fig. 2, Table 1). Inactivation of the *rpoD4* gene resulted in about a 50% decrease in amplitude of *psbAI* expression and an advance of the phasing of the peaks by ~4 hours, similar to the *rpoD3* (PCC 6803 *sigD*-type) mutant.^71^ As for *purF* expression, the period was lengthened by about 3 hours and the amplitude was decreased. In the mutant, however, the amplitude of *kaiB* expression was not significantly affected. Thus, disruption of *rpoD4* had a similar effect on circadian expression as that of *rpoD3*. The double mutant of *rpoD3* and *rpoD4* showed a shortened period of *kaiB* expression by ~1.5 hours, indicating that the *kaiB* promoter was redundantly recognized by RpoD3 and RpoD4.^71^ An immunoblot analysis showed that the RpoD4 protein level oscillates with peaks at about circadian time 24 hours under continuous light.^71^ RpoD6 is the most recently identified group 2 σ factor in PCC 7942. Thus, no functional information is available to date. In marine cyanobacteria, a DNA microarray analysis revealed that gene expression of *PMT2246* (M1-type) and *PMT0346* (M2-type) of *Prochlorococcus marinus* MIT9313 and *PMM1697* (M1-type) and *PMM1289* (M2-type) of *Prochlorococcus marinus* MED4 was up-regulated by nitrogen depletion.^102^ Furthermore, a bioinformatic analysis predicted a potential NtcA-binding site in the promoter region of *PMT2246*,^92^ suggesting that expression of the σ factor gene is controlled by NtcA, as is the case for PCC 6803 *sigE* (see section 2–2–4–1).

There is a unique cluster of group 3 σ-factors, the SigJ-type, adjoining the SigF-type (Fig. 2). In PCC 7120, Alr0277 (SigJ-type) was previously known as Sigma-37,^24^ but designated as SigJ based on the result of a phylogenetic analysis using σ factors of PCC 7120 and those of other cyanobacteria, since SigJ proteins (PCC 7120_Alr0277, PCC 7942_1784, and *Anabaena variabilis* ATCC29413_Ava3085) formed another cluster of SigF.^29^ The expression of the *Nostoc* HK-01 *sigJ* gene (ortholog of PCC 7120 *sigJ)* was significantly induced in the mid-stage of dehydration, and was upregulated before that of other σ factor genes. Subsequently, they characterized PCC 7120 SigJ under dehydration because a transformation method for cells of *Nostoc* HK-01 had not been established. It was observed that a higher-expressing transformant of the *sigJ* gene acquired desiccation tolerance. Furthermore, a genome-wide analysis with a DNA microarray showed that a comparatively large number of genes relating to polysaccharide biosynthesis were upregulated in the transformant. In accordance with the data from the microarray analysis, the amount of extracellular polysaccharide released into the culture medium was as much as 3.2-fold that released by the control cells. Thus, PCC 7120 SigJ is a key regulator of desiccation tolerance and regulates the synthesis of extracellular polysaccharide. Accommodating dehydration is important for cell survival, since drying causes cell lysis and damage to nucleic acids, proteins, and membranes.^111^,^112^ However, the *sigJ*-type gene is not conserved in most cyanobacteria, as shown in Table 1, despite its important role in acclimation. In *Nostoc* HK-01, the expression of group 2 and 3 σ factor genes except for *sigJ* was induced in the late stages of dehydration. Therefore, it seems plausible that some other type of σ factor is also involved in gene expression for adapting to dehydration.

How is there universality of gene expression and functions for respective group 2 and 3 σ factors, involving the M- and SigJ-types, among different cyanobacteria? Comprehensive and comparative (across) analyses of type σ factors among different cyanobacteria are awaited.

### 2–4. Interference of expression among group 1, 2, and 3 σ factor genes

In 2003, it was reported that a regulatory network exists among σ factors in cyanobacteria, which was discovered during an analysis of SigB expression.^39^ As mentioned for the SigB-type σ factor, PCC 6803 SigB protein decreased in response to light. The reduction was observed when chloramphenicol (inhibitor of translation) was added to the cells, however, intriguingly, it was inhibited by addition of rifampicin (inhibitor of transcription). These results implied that the down-regulation of SigB expression was achieved by *de novo* synthesized RNAs under light. Thus, subsequently, the SigB protein level was examined in group 2 σ factor knockout strains. Surprisingly, a large amount of SigB remained in the *sigC* knockout strain even 3 hours after exposure to light. Consistent with this, *sigB* transcript levels were dramatically elevated in the *sigC* knockout strain, being approximately 11 times higher than in the wild type at 0.5 hours after the shift to light. The mechanism by which SigC represses *sigB* expression remains unknown, though these results clearly indicated a regulatory network among group 2 σ factors. After that report, some groups revealed a regulatory network among group1, 2, and 3 σ factors in PCC 6803.^40^,^53^,^57^,^72^,^89^,^105^,^113^,^114^ All these studies indicated interference of expression among group 1, 2, and 3 σ factors at the level of the transcript and protein. These results mean that disruption of one σ factor essentially alters the expression of other σ factor(s). It should be noted that some results appear contradictory. The discrepancies might result from materials being prepared at different sampling times, experimental conditions, and/or strain background. More detailed experiments appear to be required.

As the expression of group 1, 2, and 3 σ factor genes is controlled by each other, *in vitro* transcription analysis is a powerful tool for demonstrating direct recognition by an σ factor of its target gene(s). How can we obtain substantial evidence for σ factor’s target genes *in vivo* without using the standard genetic approach? Chromatin immunoprecipitation (ChIP) is widely used for quantifying protein-DNA interactions in living cells.^115^ In fact, it was recently used to quantify the binding of RpaB to the *rpoD3* and *hliA* promoter regions in PCC 7942.^116^ One can also monitor the binding of σ factors in their target gene’s promoter in wild-type living cells under several growth conditions using ChIP. Thus, *in vitro* transcription and/or ChIP analyses as well as the conventional genetic approach will be useful for the characterization of σ factors in cyanobacteria.

## 3. Promoter Types

As described in section 2, a unique feature of cyanobacteria is the presence of many group 2 type σ factors. The diversity of group 2 type σ factors impacts on promoter recognition and interference with the expression of group 1 σ factors. The novel ability of a group 3 σ factor, SigF, to recognize promoters has been known in PCC 6803. Promoter types of PCC 6803 connected with RNAP and σ factors are summarized in this section (Fig. 4). Also discussed is (i) the group 1 σ factor contributing to the basal transcription from the type 1 promoter, (ii) how group 2 σ factors function in promoter recognition for transcription from type 1 and 2 promoters in replacement of the group 1 σ factor?, and (iii) a type 3 promoter which may not depend on type 1 and 2 promoters.

### 3–1. Type 1 promoters

Type 1 promoters possess both −35 (TAGACA) and −10 (TATAAT) hexamers as the σ^70^-dependent consensus sequence. The type 1 promoter is basically recognized by the group 1 σ factor under normal physiological conditions, but some group 2 σ factors are induced under stressful conditions and may replace the group 1 σ factor in RNAP whose holoenzyme can more efficiently drive transcription from the type 1 promoter. For example, the light-responsive expression of *psbA* genes, encoding the photosystem II reaction center protein D1, has been characterized in PCC 6803.^20^,^24^,^39^,^89^,^117^ The group 1 σ factor SigA can constitutively recognize the *psbA* promoters for basal transcription, as the *psbA* transcripts were still observed on knockout of the group 2 σ factors.^39^,^89^ On the other hand, the group 2 σ factor SigD contributes to the light-induced transcription of *psbA* under light or high-light conditions.^39^,^50^,^89^ Conceivably, the σ factor binding to the RNAP core enzyme changes from SigA to SigD in response to light. This concept does not contradict experimental results: the ratio of protein levels of 8 fmol SigA (/μg total protein) versus 3.5 fmol SigD (/μg total protein) against the RNAP core enzyme (α subunit, 35 fmol/μg total protein) changed to 8 fmol SigA versus 8 fmol SigD under light-induction in PCC 6803 cells.^24^,^39^ In addition, the group 2 σ factor SigE, whose rhythmic peak of protein expression occurs at 24-hour intervals according to the upcoming night, also contributes to the light-induced transcription of the photosynthesis-related genes *psbA*, *cpcBACD*, *petBD*, and *psaAB.*^39^,^89^ Experimental data implies that switching from SigA (or SigD) to SigE among the group 1 and 2 σ factors is also involved in the control of circadian rhythm. Taking these findings into consideration, group 1 and 2 σ factors coexist and may coordinate transcription from the type 1 promoter^18^,^20^,^39^,^40^,^89^ (Fig. 4, Top). Although we focused on light-responsive genes, the type 1 promoters may be a major group among gene promoters. Given the coexistence and interference among group 1 and 2 σ factors, the replacement of σ factors in response to environmental stress observed in some cases of transcription from type 1 (and type 2, see the next section) promoters may be a general mechanism in cyanobacteria. The type 1 sequences as single or multiple promoters are involved in transcription for cell growth and survival. Thus, the sequence similarity of the type 1 promoter has been well characterized in not only PCC 6803 but also other cyanobacteria.^43^,^44^,^118^,^119^

### 3–2. Type 2 promoters

Type 2 promoters possess only the −10 hexamer or plus enhancer-motif sequences associated with transcriptional activator proteins that may compensate for the lack of function of the −35 hexamer. Because samples for type 2 promoters are limited (Fig. 4), we discuss a representative case. The PCC 6803 *glnB* P2 promoter (PglnB-54/-53) has only the −10 hexamer and a GTA-N8-TAC motif for binding by NtcA. This motif confers an enhancement of transcription.^40^,^58^ SigB and SigC (or SigE) contribute to the transcription from PglnB-54/-53 with a σ factor, changing their population and/or activity in a growth-phase-dependent manner under nitrogen-deprived conditions. This also indicates that multiple group 2 σ factors take part in the transcription from the type 2 promoter together with the group 1 σ factor, SigA, as in the case of the type 1 promoter. In PCC 6803, RNAP-SigA can recognize *in vitro* the PglnB-54/-53 type promoters, PglnA-48/-47, Pamt1–142, and PglnN-32, in the presence of NtcA, but exhibit little or no activity without NtcA.^40^ In another case, the promoter of dark-inducible *lrtA* is assigned to a subclass of type 2 having only the −10 hexamer with extended TGTGn and GC sequences,^20^ which exhibit similarity to those directly recognized by region 2.5 of *E. coli* RpoD and RpoS, respectively. The selectivity of SigB for the *lrtA* promoter was directly confirmed *in vitro* by the redundancy of other group 1 and 2 σ factors.^20^ PpsbD-14 and PsigB-64 are assigned to another subclass of type 2 possessing only the −10 hexamer. Although the selectivity of group 1 and 2 σ factors for the PpsbD-14 and PsigB-64 promoters has not been elucidated, a model case using a mutagenized *psbA* promoter (PpsbAmt), whose sequence possesses only the −10 hexamer as an artificial type 2 promoter, has been reported for SigA and SigD.^20^ SigD can recognize PpsbAmt *in vitro* but SigA can not, indicating that the −35 hexamer is essential for RNAP-SigA in *psbA* transcription. Therefore, it seems that the transcription is driven by RNAP-SigA on the type 2 promoter. In other words, the group 2 σ factors may change with race, population, and/or activity with (or without) regulators under stress, and contribute to the transcription from the type 2 promoter in the presence of SigA. How do the group 2 σ factors exercise promoter recognition for transcription from type 1 and 2 promoters in replacement of the group 1 σ factor? As mentioned in section 2, there are several possibilities for the replacement of group 1 with group 2 σ-factors: (i) the group 2 σ factors are induced and increase in population under stress, (ii) co-activators and/or substances (e.g. ppGpp) are expressed under stress and enhance the binding of the group 2 σ factor to the core RNAP enzyme, (iii) the group 2 σ factors are modified at the post-translational level and are activated, and (iv) the group 1 σ factors are inactivated by anti-σ-factors (reported as RSD protein in *E. coli*)^77^,^78^ and the group 2 σ factors are activated.

### 3–3. Type 3 promoters

Stringent promoter recognition by PCC 6803 SigF was reported recently.^21^ Nucleotide sequences of the type 3 promoters (*pilA1*, *pixJ1*, *sll0837*, and *sigF)* are distinct from those of the type 1 and type 2 promoters. Transcription from type 3 promoters may also be independent of group 1 and group 2 σ factors, as in the case of the *pilA1* promoter recognized by SigF. Actually, *in vitro* transcription analyses demonstrated that the group 3 σ factors could not recognize the type 1 promoters.^21^,^24^ It remains to be clarified whether all group 3 σ factors contribute to transcription from type 3 promoters. Influences of expression at the level of the transcript and protein among group 1, 2, and 3 σ factor genes in PCC 6803 have been reported recently.^53^,^57^,^105^,^113^,^114^ Further identification and characterization of other promoters recognized by SigF will help to clarify the issue. Functions of and promoter recognition by PCC 6803 group 3 σ factors, SigG, SigH, and SigI, have been unclear to date. Analyses using *in vitro* transcription systems together with knockout strains may also be useful for the identification and characterization of novel promoters recognized by group 3 σ factors.

## 4. Conclusion and Future

Transcription in cyanobacteria is driven by the RNAP core enzyme with heterogeneous σ factors which are assigned to groups 1, 2, and 3. The evolutional diversity in terms of the population and function of cyanobacterial group 2 σ factors is unique. The network for gene expression of σ factors influences each other. Coordination among group 1 and 2 σ factors seems to contribute to the sensing of environmental changes or fine tuning for transcription from type 1 or 2 promoters. On the other hand, a stringent control for promoter recognition has been reported for the type 3 promoter. Functional analyses of structure and amino acid sequence, conferring differences in promoter selectivity, are required. Moreover, the characterization of unique structures in core enzyme subunits in RNAP and identification of transcription factors (e. g. response regulators in two-component systems) interacting with RNAP are also awaited for insights into cyanobacterial transcription.

## Figures and Tables

**Table 1:** Classification of σ factors in representative cyanobacteria and *E. coli.*

Group		Type	PCC 6803	NIES-843	PCC 7002	PCC 7942	BP-1	PCC 7120	Group		*E. coli*
1		A	SigA (Slr0653)	MAE54470	SigA (SYNPCC7002_A2014)	RpoD1 (Synpcc7942_0649)	SigA (TII0617)	SigA (AII5263)	1		RpoD (BAE77118)
2		B	SigB (SII0306)	MAE14230	SigB (SYNPCC7002_A1202)	RpoD2 (Synpcc7942_1746)	SigB (TII0831)	SigB^a^ (AII7615) SigB2 (Alr3800) SigB3^a^ (AII7608) SigB4^a^ (AII7179)	2		RpoS (BAE76818)
		C	SigC (SII0184)	MAE09350	SigE (SYNPCC7002_A0270)	RpoD5/SigC (Synpcc7942_1849)	SigC (Tlr0499)	SigC (AII1692)			
		D	SigD (SII2012)	MAE45250	SigD (SYNPCC7002_A1832)	RpoDS (Synpcc7942 _0672)	SigD (Tlr0264)	SigD (Alr3810)			
		E	SigE/RpoDV-2 (SII1689)	MAE52900	SigC (SYNPCC7002_A0364)	-	-	SigE (Alr4249)			
		Others (M)	-	-	-	RpoD4 (Synpcc7942_0569) RpoD6 (Synpcc7942_1557)	-	-			
3		F	SigF (Slr1564)	MAE52920	SigF (SYNPCC7002_A1924)	Synpcc7942_1510	SigF (Tlr0738)	SigF (AII3853)	3		RpoF (BAA 15742)
	Ecf-like	G	SigG (Slr1545)	MAE02820	SigG (SYNPCC7002_A1970)	Synpcc7942_1923	SigG (Tlr2109)	SigG (Alr3280)			RpoH (BAE77832)
		H	SigH (SII0856)	MAE49220	SigH (SYNPCC7002_A2111)	_	SigH (Tlr0317)	-			
		I	SIgI (SII0687)	-	-	Synpcc7942_2004	-	Sigl (AII2193)		Ecf	RpoE (BAE76749)
		J	-	-	-	SigJ (Synpcc7942_1784)	-	SigJ (Alr0277)			Fecl (BAE78284)
RpoN		-	-	-	-	-	-	RpoN	RpoN (BAE77246)

For cyanobacteria, the named gene product with the gene number in CyanoBase or the gene number in CyanoBase is indicated. For *E. coli* (K-12 strain W3100), the named gene product with the GenBank accession number is indicated.

a Encoded on plasmids.
